# Interaction of the Mouse Polyomavirus Capsid Proteins with Importins Is Required for Efficient Import of Viral DNA into the Cell Nucleus

**DOI:** 10.3390/v10040165

**Published:** 2018-03-31

**Authors:** Irina Soldatova, Terezie Prilepskaja, Levon Abrahamyan, Jitka Forstová, Sandra Huérfano

**Affiliations:** Department of Genetics and Microbiology, Faculty of Science, Charles University, Vinicna 5, 12844 Prague 2, Czech Republic; soldati@natur.cuni.cz (I.S.); svoboter@natur.cuni.cz (T.P.); levon.abrahamyan@umontreal.ca (L.A.); jitka.forstova@natur.cuni.cz (J.F.)

**Keywords:** mouse polyomavirus, trafficking into the nucleus, importin β1, capsid proteins, nuclear localization signal

## Abstract

The mechanism used by mouse polyomavirus (MPyV) to overcome the crowded cytosol to reach the nucleus has not been fully elucidated. Here, we investigated the involvement of importin α/β1 mediated transport in the delivery of MPyV genomes into the nucleus. Interactions of the virus with importin β1 were studied by co-immunoprecipitation and proximity ligation assay. For infectivity and nucleus delivery assays, the virus and its capsid proteins mutated in the nuclear localization signals (NLSs) were prepared and produced. We found that at early times post infection, virions bound importin β1 in a time dependent manner with a peak of interactions at 6 h post infection. Mutation analysis revealed that only when the NLSs of both VP1 and VP2/3 were disrupted, virus did not bind efficiently to importin β1 and its infectivity remarkably decreased (by 80%). Nuclear targeting of capsid proteins was improved when VP1 and VP2 were co-expressed. VP1 and VP2 were effectively delivered into the nucleus, even when one of the NLS, either VP1 or VP2, was disrupted. Altogether, our results showed that MPyV virions can use VP1 and/or VP2/VP3 NLSs in concert or individually to bind importins to deliver their genomes into the cell nucleus.

## 1. Introduction

Mouse polyomavirus belongs to the *Polyomaviridae* family, a group of tumorigenic non-enveloped double stranded DNA viruses. Polyomaviruses (PyVs) infect different vertebrates including humans; however, new subtypes recently found in invertebrates have been described [1]. The number of newly discovered mammalian PyVs has increased dramatically in recent years. Simian virus 40 (SV40) and MPyV have served as model viruses for many years and are by far the best studied. Nonetheless, several gaps in our understanding of the mechanisms of their replication cycle (e.g., genome delivery into the cell nucleus or virion assembly) exist and remain to be elucidated. The capsid of MPyV is composed of 360 molecules of the VP1 protein organized into 72 capsomeres, VP1 pentamers, forming a T7 icosahedral surface lattice. Each capsomere contains one molecule of the minor capsid protein, either VP2 or its shorter variant, VP3 [2,3]. Capsomeres—complexes of five VP1 molecules with one VP2 or VP3 molecule are assembled shortly after protein synthesis in the cytoplasm and then they are imported to the nucleus for virion assembly. The complexes are formed even during co-expression of proteins out of context of infection [4,5,6,7]. The capsid encloses the MPyV genome, which is organized into a minichromosome composed of a supercoiled circular double-stranded 5.3 kb DNA molecule associated with host cell histones 2A, 2B, 3, and 4 [8]. At the first stage of productive infection, the polyomavirus binds ganglioside receptors [9] at the cell surface and becomes internalized into smooth monopinocytic vesicles [10,11,12]. Then, the virus is sorted into the early and late endosomes. Indeed, infection requires the acidification of endosomes as raising the endosomal pH markedly reduces viral infectivity [13,14]. The virus is then transported to the endoplasmic reticulum (ER). For virus replication, PyV genomes need to be transported into the cell nucleus. Based on electron microscopy analyses, early studies suggested that SV40 [15] and MPyV [16] enter the nucleus by fusion of vesicles carrying virions directly with the nuclear envelope, bypassing nuclear pores. The possibility of direct penetration of the virus from the ER to the cell nucleus through inner nuclear membrane has also been suggested [17].

More recent studies, performed so far with SV40, JC polyomavirus (JCPyV), and BK polyomavirus (BKPyV) strongly support the hypothesis that viruses translocate from the ER to the cell cytosol and use the canonical route of DNA trafficking into the nucleus mediated by importins [18,19,20]. In the ER, polyomaviruses undergo rearrangements that involve the reduction and/or isomerization of disulfide bonds of viral capsid proteins [21]. Conformational changes in the capsid lead to the exposition of the hydrophobic proteins VP2 and VP3 [22,23,24,25]. The modified “hydrophobic” virus interacts with the ER membrane and with the ER translocon related proteins [21,25,26,27]. Tsai et al. showed by using a modified cell fractionation method that a partially modified, but still large viral particle composed of VP1, VP2/3, and DNA exited the ER to the cytosol [28]. Geiger et al. demonstrated that the subpopulation of chemically labeled SV40 was remodeled in the ER and suggested that the remodeled virus was able exit to the cytosol [23].

In cytosol, the trafficking of proteins or their complexes into the cell nucleus is mediated by the interaction of their NLS (nuclear localization signal) with α and β importins. Importin α recognizes and interacts with NLS and then associates with importin β1. The trimeric importin α-importin β-NLS complex translocates into the nucleus [29]. All three capsid proteins of MPyV and SV40 contain NLSs [30,31,32]. Nakanishi et al. described that V1 and VP3 proteins of SV40 bind to importins in vitro, but that only interaction between VP3 and importins was required for infection [33]. In agreement, Bennet et al. showed that the disruption of NLS sequences of the minor capsid proteins VP2 and VP3 of BKPyV decreased infectivity to half [20]. On the other hand, Qu et al. reported that the nuclear translocation of JCPyV virus like particles, composed of DNA and VP1 only, was dependent on the interaction between the NLS motif of VP1 and cellular importins [19].

To better understand the relevance of the interaction of importins with the capsid proteins VP1, VP2, and VP3 in polyomavirus infection, we followed the interaction of importins with MPyV during early times post infection by using immunoprecipitation and the proximity ligation assay (PLA). In addition, we prepared various mutated viruses and plasmids for the expression of capsid proteins carrying mutations disrupting or enhancing the NLS character of the viral capsid proteins. Mutated viruses were used for infectivity assays and mutant proteins for the analysis of nuclear sorting using confocal microscopy.

## 2. Materials and Methods

### 2.1. Cell Lines, Plasmid DNA and DNA Transfection

Mouse fibroblasts, 3T6 (ATCC; Manassas, VA, USA; CCL-96), were used for viral production and infection and NIH 3T3 (ATCC; CRL-1658) were used for transfection. Cells were grown at 37 °C in a humidified incubator with a 5% CO_2_ atmosphere, using Dulbecco’s Modified Eagle’s Medium (DMEM) (Sigma-Aldrich, St. Louis, MO, USA) supplemented with 5% fetal bovine serum (Invitrogen, Carlsbad, CA, USA) and 4 mM l-glutamax (Sigma-Aldrich). Plasmid DNA was prepared using a JETSTAR Plasmid Purification Kit (Genomed, Löhne, Germany). Plasmid concentration and purity were determined by nanodrop. Transfections were performed by a nucleofector™ device, using an amaxa-kit V (Lonza, Basel, Switzerland) according to the manufacturer’s instructions.

### 2.2. Viral Infection

Cells were seeded on 13-mm glass coverslips in 24-well or 3-cm plates and were grown for the time periods indicated in each experiment. At the day of infection, cells were washed and incubated with wt MPyV or mutants diluted in serum-free medium for 1 h on ice (co-immunoprecipitation and proximity ligation assay) or at 37 °C. After virus adsorption, medium containing serum was added and cells were incubated at 37 °C. The infection start was measured from the time of cell transfer to 37 °C.

### 2.3. Negative Staining

Viral samples for negative staining were processed by placing a drop of the sample on a carbon-coated copper, furthermore, the grid was stained with 2% phosphotungstic acid. The samples were observed with a JEOL JEM (Akishima, Tokyo, Japan) 1200EX electron microscope operating at 80 kV. Fine structure measurements/observations were performed using a Veleta camera and iTEM 5.1 software (Olympus Soft Imaging Solutions GmbH, Münster, Germany).

### 2.4. Transmission Electron Microscopy

Transfected cells were washed in phosphate-buffered saline, fixed with 3% glutaraldehyde in 0.1 M cacodylate buffer, post-fixed with 1% osmium tetroxide, dehydrated through an increasing ethanol series and flat-embedded in Agar resin. Thin sections (70 nm) were cut on a Reichert-Jung Ultracut E ultramicrotome and stained using uranyl acetate and lead citrate. Sections were examined and photographed using a JEM-1011 electron microscope (JEOL Akishima, Tokyo, Japan). Fine structure measurements/observations were performed using a Veleta camera and iTEM 5.1 software (Olympus Soft Imaging Solutions GmbH).

### 2.5. Indirect In Situ Immunofluorescence

Cells growing on coverslips were fixed with 4% paraformaldehyde for 15 min, then cells were permeabilized with 0.5% Triton X-100 in 1× PBS for 5 min, followed by three times washing in 1× PBS. After that, cells were blocked with 0.25% bovine serum albumin and 0.25% porcine skin gelatin in PBS. Immunostaining with primary and secondary antibody was carried out for 1 h and 30 min, respectively, with extensive washing in PBS after each incubation.

### 2.6. Co-Immunoprecipitation and Cross-Linking

The importin β1 antibody or nonspecific IgG control antibody was first bound to protein G dynabeads (Invitrogen) in 0.02% Tween-20 in PBS. The mixture was incubated on a mixing rotor at 4 °C for 30 min. After incubation, the beads were separated and washed with 0.02% Tween-20. The antibody was crosslinked to the beads by using a disuccinimidyl suberate (DSS) crosslinker (Fisher Scientific, Waltham, MA, USA). Next, 230 µL of cell lysate (from the 3-cm plate) was prepared in modified RIPA Buffer (without SDS), supplemented with a protease inhibitor cocktail (Roche, Penzberg, Upper Bavaria, Germany). Aliquots (30 µL) of whole cell lysates were saved as input controls. The rest (200 µL) was added to beads-antibody complexes and incubated with rotation overnight at 4 °C. After incubation, the beads (containing antibody-protein complexes) were washed. Beads were divided into two fractions: fraction one was used for suspension in a Laemmli buffer and analyzed using SDS-PAGE (10% gel) followed by western blot and the second fraction was used for DNA isolation.

### 2.7. PCR Detection of DNA Isolated from Immunocomplexes

Samples containing antibody-antigen complexes bound to dynabeads were resuspended in 500 µL of PBS buffer containing Proteinase K (100 µg/mL) (Roche) and 5 µL of 10% SDS. Next, samples were heated at 55 °C for 30 min and DNA was isolated by treatment with phenol-chloroform and precipitated with acetate/ethanol overnight at 4 °C. After precipitation, the samples were centrifuged, the precipitate was dried and resuspended in PCR grade water. The samples were subjected to PCR for amplification of a fragment of MPyV genomic DNA. The primers used for the reaction were: forward 5′-TGATTCTTCGGGATTTG-3′ and backward 5′-GTGGCGTTGCATT-3′. The expected product should be 250 bp long.

### 2.8. Proximity Ligation Assay

We used a Duolink kit (Sigma-Aldrich) according to the manufacturer’s instructions. The probes used were anti-rabbit plus (DUO92002) and anti-mouse minus (DUO92004). Infected cells were fixed and incubated with two combinations of antibodies: mouse antibody against VP1 with rabbit antibody against importin β1, or mouse antibody against VP1 with rabbit antibody against VP1. The later combination was used as the control. After washing, cells were incubated with secondary anti-mouse and anti-rabbit antibodies tagged with PLA oligoprobe. Then, the ligation reaction was performed. For detection of ligation products, in situ PCR was carried out and the products visualized by the hybridization of fluorescently labeled oligonucleotides (λex = 594 nm and λem = 624 nm). The signal was detected by fluorescence microscopy.

### 2.9. Agarose Gel Electrophoresis

Samples for DNA detection were run in 0.8% agarose gel stained by GelRed TM Nucleic Acid Gel Stain (Biotium, Fremont, CA, USA) according to the standard procedure.

### 2.10. Virus Production, Isolation and Quantification

pMJG plasmid, containing the entire genome of MPyV strain A3 (opened and inserted into the bacterial plasmid in the unique EcoRI site), was used as a source of wt MPyV genome. Derivatives of pMJG: VP1 K6Q-VP2/VP3 K315A; VP1 K6Q-VP2/VP3 K314A-K315A, and VP1 K6Q-S7R-G8R-VP2/VP3 K314A-K315A-R317A, were produced by a GENEART^®^ Site-Directed Mutagenesis System kit (Invitrogen), according to the manufacturer’s instructions. To prepare wt and mutated MPyV viruses, genomes were excised from the plasmids with EcoRI and circularized. Ligation mixtures were used for the transfection of 3T6 cells by nucleofection. Transfected cells were incubated per 6–8 days and then the virus was isolated by CsCl gradient [34]. Viruses were subjected to hemagglutination assay using guinea pig erythrocytes as previously described [34]. The HA titre was defined as the reciprocal of the highest virus dilution showing haemagglutination. The tittres of wt virus and mutant viruses 1 and 3 varied in different isolations within the order 10^13^ hemagglutination units (HU)/mL and the titter for the mutant virus 2 was within the order 10^10^ HU/mL.

### 2.11. NLS Sequence Analysis

Sequences of the VP2/VP3 and VP1 capsid proteins of wt, mutant 1, mutant 2, and mutant 3 of MPyV were analyzed using the NucPred program, available online [35].

### 2.12. Viral Genome Quantification

Viral particles were cleaned from extracellular DNA by treatment with DNase I (0.4 U/µL) to remove residual extracellular DNA. After DNase inactivation, capsids were disassembled by 

proteinase K (Roche) and DTT (Sigma) treatment and viral DNA was extracted by phenol-chloroform, followed by ethanol precipitation according to the protocol previously described [36]. Viral DNA was quantified by real-time PCR assay using iQ™ SYBR^®^ Green Supermix (Bio-Rad Laboratories, Hercules, CA, USA) detection and amplified using primers set to fragments of VP1 and LT genes: VP1 5′-GCAAGAAGGCGACGAC-3′ and 5′-TGGCCTCCCTCATAAGT-3′ and LT 5′-GCTGACAAAGAAAGGCTGCT-3′ and 5′-AGCCGGTTCCTCCTAGATTC-3′. Thermal cycling was performed in a Light Cycler 480 II from Roche. Values of samples were obtained by comparison of samples with a standard curve of known viral DNA concentrations.

### 2.13. SDS-PAGE and Western Blot Analysis

Cells were harvested and washed with phosphate-buffered saline, then resuspended in ice-cold cell lysis buffer (10 mM Tris/HCl, pH 7.4, 1 mM EDTA, 150 mM NaCl, 1% Nonidet P-40, 1% sodium deoxycholate, 0.1% SDS) supplemented with a protease inhibitor cocktail (Complete Mini EDTA free, Roche). Cell lysis was carried per 20 min on ice. Cell debris was removed by centrifugation. Proteins were resuspended in Laemmli buffer and applied to 10% acrylamide gel for SDS-PAGE. The samples were blotted to a nitrocellulose membrane and detected by immunostaining with selected antibodies. For detection of minor proteins in samples after co-immunoprecipitation we used ultra-sensitive enhanced chemiluminescent substrate supersignal west femto (Thermo Fisher Scientific, Waltham, MA, USA).

### 2.14. Introduction of Mutations into Individual Proteins VP1 and VP2

The plasmid pVP1 encoding for MPyV VP1 from LID strain [37] and ph2pΔG encoding for MPyV VP2 from LID strain [38] were used to introduce mutations in the NLS coding sequences of the capsid proteins VP1 and VP2. Mutations were introduced by using a GENEART^®^ Site-Directed Mutagenesis System (Invitrogen) kit, according to the manufacturer’s instructions. The following protein variants were produced: VP1/K6Q, VP1/K6Q-S7R-G8R, VP2/K315A, VP2/K314A-K315A, and VP2/K314A-K315A-R317A.

### 2.15. Antibodies

Mouse monoclonal anti-VP2/3, rabbit polyclonal anti-VP1 antibody, (produced in our laboratory), mouse monoclonal anti-VP1, rat monoclonal anti-large T antigen (LT) (provided by B. E. Griffin, Imperial College of Science, Technology and Medicine at St. Mary’s, London, UK), mouse monoclonal anti-importin β1 antibody (Fisher Scientific), rabbit anti-importin β1 antibody (Bioss Antibodies, Woburn, MA, USA), normal mouse IgG (Upstate Biotechnology, Lake Placid, NY, USA), normal rabbit IgG (Upstate Biotechnology), Alexa Fluor^®^ 488 donkey anti-mouse IgG (Thermo Fisher Scientific), Alexa Fluor^®^ 488 goat anti-rat IgG (Thermo Fisher Scientific), Alexa Fluor^®^ 546 donkey anti-rabbit IgG (Thermo Fisher Scientific), goat anti-rabbit IgG-HRP (Bio-Rad), and goat anti-mouse IgG-HRP (Bio-Rad).

## 3. Results

### 3.1. Importin β1 Binds Virions of MPyV at Early Times Post Infection

**Figure 1:**
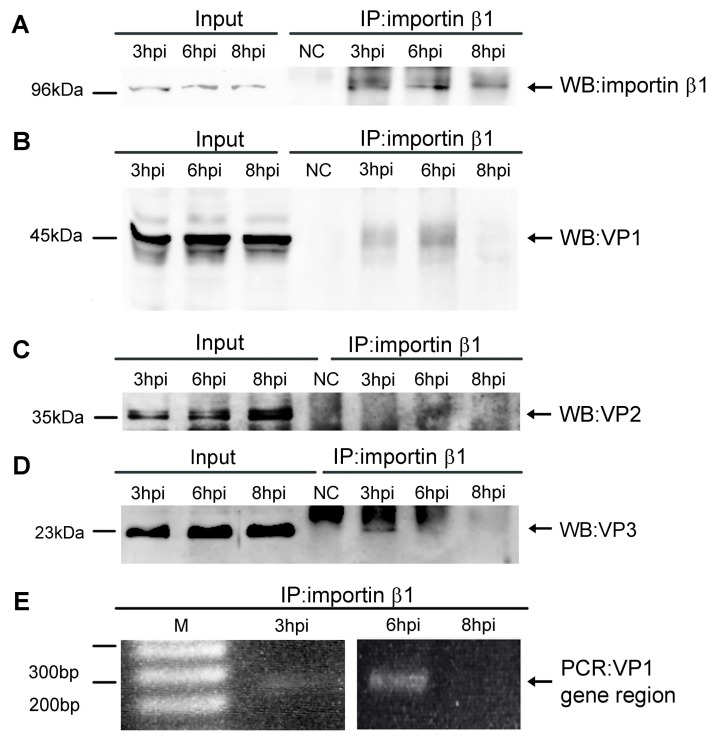
In vivo interactions between importin β1 and viral particles at early times post infection. Western blots of 3T6 cell lysates (labeled as Input) or complexes precipitated through antibody against importin β1 (labeled as IP) were performed with antibody against importin β1 (**A**), VP1 (**B**) or VP2/VP3 (**C**,**D**). As a negative control (NC), samples precipitated through nonspecific anti-mouse IgG were used. The viral DNA was isolated from complexes and amplified by PCR at indicated times post infection (**E**).

First, to evaluate the ability of MPyV virions to bind importin β1 at different times post-infection, a co-immunoprecipitation assay was performed. Cells were infected with 2 multiplicity of infection (MOI) and harvested at 3, 6, and 8 h post infection (hpi). Cell lysates were prepared and immunoprecipitation was performed by using a monoclonal antibody against importin β1 or non-specific IgG antibody (as the negative control). Then, we followed the presence of the major capsid protein, VP1, or importin β1 in immune complexes by western blot analysis (Figure 1A,B).

Complexes containing both the viral VP1 capsid protein and importin β1 were detected at 3 and 6 hpi, while at 8 hpi, no VP1 was co-immunoprecipitated with the antibody against importin β1 (Figure 1A,B). At the same time, the level of VP1 capsid protein precipitated at 3 hpi was lower than at 6 hpi. In the immunoprecipitated complexes, the presence of the minor capsid proteins VP2 and VP3 was verified using specific antibody against VP2/VP3 (Figure 1C,D). The detection of the minor proteins was performed using enhancer due to low proportion of the protein in virions. We detected VP3 at 3 and 6 hpi while we detected VP2 only at 6 hpi. This likely due to the ratio of VP2/VP3 in virions. The control (nonspecific anti-mouse IgG) did precipitate neither the viral proteins nor importin.

In addition, the presence of viral DNA in immune complexes was verified by PCR amplification of a fragment of viral DNA, isolated from the immunocomplexes using primers specific for sequences from the VP1 gene region. The presence of MPyV DNA was detected at 3 and 6 hpi, but not at 8 hpi (Figure 1E). Thus, the amounts of viral DNA at studied time points correlated with the levels of immunoprecipitated VP1. These results indicate that MPyV virions are accessible for importins at early times post infection.

**Figure 2:**
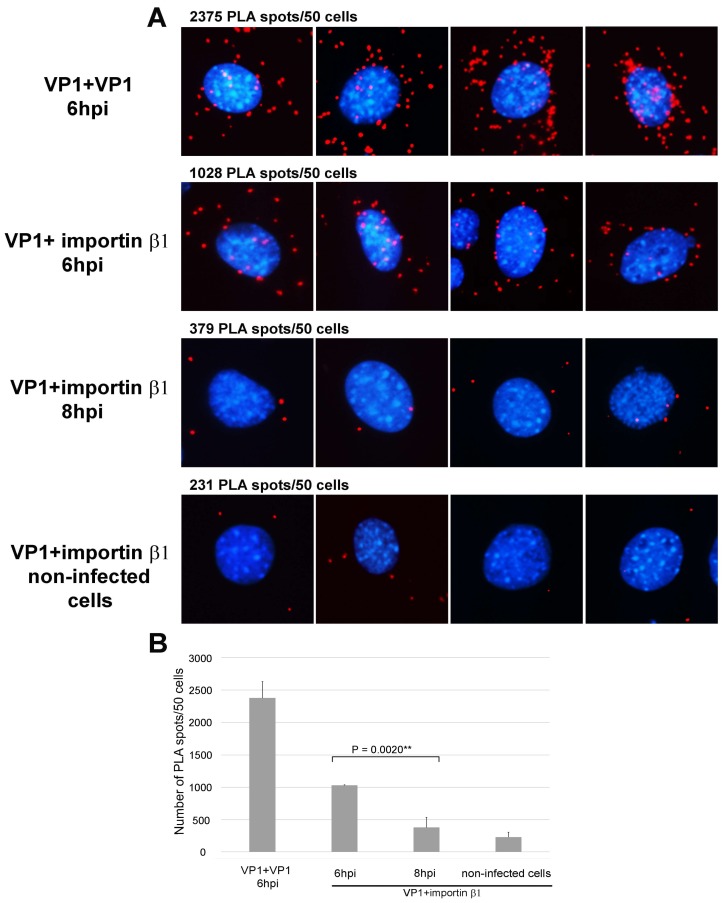
Proximity ligation assay of VP1 and importin β1 at 6 and 8 hpi. (**A**) PLA assay was performed on 3T6 cells infected with MPyV (200 virus particles per cell), using primary mouse and rabbit antibodies against VP1 or mouse antibody against VP1 and rabbit antibody against importin β1. Next, the oligoprobes tagged anti-mouse and anti-rabbit antibodies were used. Red spots represent the products of amplification after oligonucleotide ligation. DNA was stained by DAPI. As controls, the infected cells were stained with two antibodies (mouse and rabbit) against VP1 and non-infected cells were stained with anti-VP1 and anti-importin β1 antibodies. At the top of each image, the average numbers of the PLA spots quantified in three independent experiments are presented (for each experiment 50 cells were analyzed). The pictures were taken 20× magnification; (**B**) The graph represents the mean values of three independent experiments ± SD. Samples were compared by the Student’s *t*-test. *p* values are given and asterisks represent statistically significant differences (** *p* ≤ 0.01).

To confirm and also quantify the interaction between the virus and importin β1, we used the PLA. PLA is a very sensitive technique that enables the visualization of protein-protein interaction in cells. PLA fluorescent signal/PLA spots are generated only if two antibodies targeted proteins are within a distance of less than 40 nm [39,40]. For this experiment, cells growing on coverslips were infected with a low number of viral particles (200 particles per cell) and fixed at 6 or 8 hpi. PLA was performed as indicated in the Materials and Methods section. For this assay, we used a mouse antibody against the VP1 protein and a rabbit antibody against importin β1. The number of PLA spots per cell were quantified in three independent experiments in 50 cells per experiment. Figure 2 shows representative pictures of the PLA spots (Figure 2A) and a graph of quantitative analysis (Figure 2B). As a control of the PLA assay, we used two anti-VP1 specific primary antibodies from different species: mouse and rabbit. The number of spots indicating a close proximity of virions and importin β1 per 50 cells was more than 1000 at 6 hpi, while the number of spots observed at 8 hpi decreased to 36%. The background number of PLA spots observed in non-infected (control) cells was about 230 spots per 50 cells. Altogether, our results show that the binding of the MPyV to importins is time dependent, with maximal binding occurring at 6 hpi.

### 3.2. Mutagenesis Design of the Viruses Carrying Mutations in NLS Sequences of VP1 and VP2/VP3 Genes

The stretches of amino acids (aa) containing NLS sequences of MPyV structural proteins have been previously described. By intragenic deletions, Chang et al. found that the truncation of the first 11 *N*-terminal aa (M1APKRKSGVSK11) of VP1 prevented its delivery into the cell nucleus [41]. Similarly, the deletion of the 12 *C*-terminal aa (E308EDGPQKKKRRL319) common for VP2 and VP3 caused the relocation of the proteins from the cell nucleus to the cytoplasm [31]. These aa stretches in both VP1 and VP2/VP3 proteins are enriched by basic aa Lysine (K) or Arginine (R). Importantly, the basic aa are crucial for importin recognition of the proteins [29]. To confirm the relevance of the importin-mediated transport of MPyV via nucleopores, we attempted to create virus mutants with decreased or recovered affinity of VP1 and VP2/3 capsid proteins to importins by substituting or introducing basic aa into the NLS sequence of the capsid proteins and analyze the impact on virus delivery into the cell nucleus.

Strong restrictions for the design and preparation of mutants had to be taken in account. As the *N*-terminus sequence of VP1 and *C*-terminal sequence of VP2/3 genes partially overlap (by 33 nucleotides), mutations in the NLS of the minor capsid proteins affect the NLS of VP1 and vice versa [42]. The design of mutants was based on a minimal number of substitutions and on the score value of the sequences analyzed by the nuclear localization prediction software NucPred. The NucPred program, unlike other nuclear localization prediction programs, does not use predictions based on known predefined NLS patterns. Instead, it uses a machine-learning technique that automatically develops computer programs in artificial evolution of biological processes. The evolved predictors incorporate multiple regular expressions which are matched against the input (aa) sequences. For interpretation of results, proteins with a score between 0.10 and 1 are predicted to spend some time in the nucleus. The higher score value, the higher probability that a protein is truly nuclear. In detail, proteins with score 0.8 were shown to be nuclear in 93% of cases, while proteins with score 0.1 spent some time in the nucleus only in 45% of cases [35].

**Figure 3:**
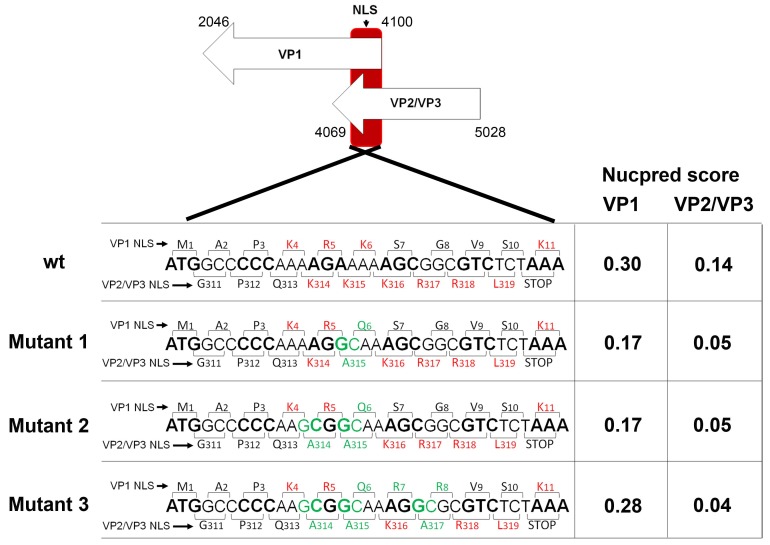
Mutagenesis design. Wild type MPyV overlapping coding sequences for VP1 and VP2/3 genes are displayed with the corresponding aa at the top of the nucleotide triplet for VP1 or at the bottom of the nucleotide sequence for VP2/3 (brackets show the codons). Positions of aa residues are given for VP1 (top) and VP2 (bottom) proteins, respectively. For better differentiation, codons for VP1 are alternate in bold and not bold letters. Mutants 1, 2, and 3 are also presented. For better display, the basic aa (K and R) are shown in red and the mutated nucleotides and corresponding aa are shown in green. NucPred score values for NLS of VP1 and VP2/3 capsid proteins of the wt or mutated variants are presented on the right.

In total, three virus mutants were proposed: mutants 1 and 2 that have mutations expected to negatively affect the NLS character of both the VP1 and VP2/VP3 proteins, and the mutant virus 3, carrying mutations expected to negatively affect the NLS character of VP2 and VP3 proteins, while preserving (and even enhancing) the NLS character of the VP1 protein. In detail, mutant 1 had one substitution in VP1 (lysine (K6) by glutamine (Q6)) and one substitution in VP2/VP3 (lysine (K315) by alanine (A315)). Mutant 2 had the same one substitution in VP1 (K6Q) and two mutations in VP2/VP3 (K314A, K315A). Mutant 3 carried three mutations in VP2/VP3 (K314A, K315A, and K317A) and in VP1, in addition to the mutation appearing in the mutants 1 and 2 (K6Q), two more arginines were introduced into the NLS sequence (S7R and G8R) to restore NLS function. Figure 3 shows the overlapping region of the wt VP1 and wt VP2/VP3 genes and the details of the mutagenesis design. In the figure, the codons for VP1 and VP2/VP3 are shown by brackets with corresponding aa sequences at the top (VP1) and at the bottom (VP2/VP3). Mutations are labeled in green while basic aa (K and R) are shown in red. Mutated NLS sequences were analyzed by NucPred (Figure 3 right). The score values were in agreement with the proposed mutations. Thus, the substitutions of basic aa with alanines (in NLS of VP2/3) or glutamine (in NLS of VP1) decreased the score of the proteins while the introduction of basic aa into the NLS of VP1 of mutant 3 increased the NucPred score almost to the value of the wild type VP1.

### 3.3. Mutant Virus Production and Characterization

**Figure 4:**
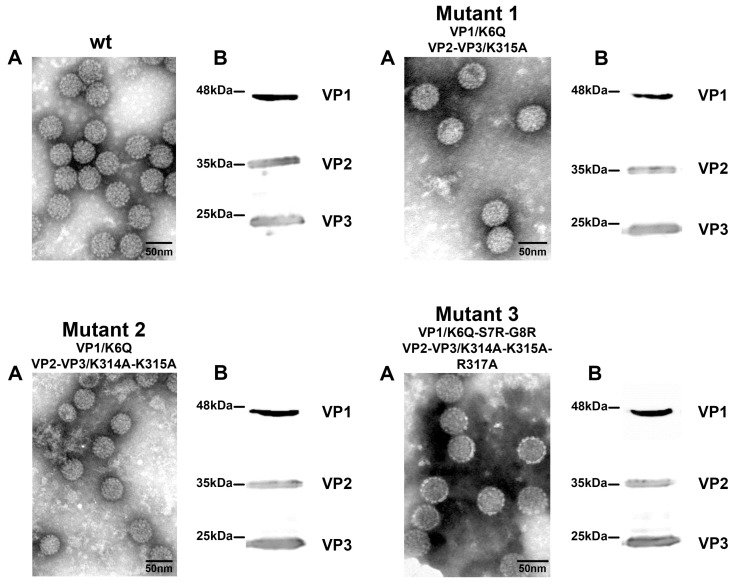
Characterization of the mutated viruses. The wt virus or mutant viruses 1, 2, and 3, isolated from transfected cells were contrasted by phosphotungstic acid and visualized by electron microscopy (Bars = 100 nm) (**A**); Presence of the capsid proteins was detected by Western blot using specific antibody against VP1 and VP2/3 (**B**).

The designed mutations (Figure 3) were introduced in the pMJG plasmid containing the entire genome of the MPyV strain A3 using the Invitrogen GENEART^®^ Site-Directed Mutagenesis System kit. The mutated genomes were transfected into 3T6 cells and, after six days, the virus was isolated by CsCl density gradients. Viral fractions were characterized by electron microscopy, Western blot for detection of the structural proteins and by genome sequencing (Figure 4 and Figure S1). The analysis showed that mutations did not affect the viral assembly and the shape of the mutant viruses. The morphology of mutants was similar to those of the wt virus (Figure 4A). Western blot analysis revealed that the electrophoretic mobilities of the mutated VP1 and both mutated minor capsid proteins corresponded to those of the wt protein counterparts and that incorporation of the minor capsid proteins into virions was not affected by mutations (Figure 4B). To verify the mutations, as well as to detect possible revertants, we sequenced genomic DNAs isolated from virus preparations. Sequencing data displayed a homogenous population of the viruses and confirmed the presence of designed mutations (Figure S1).

### 3.4. Amino Acid Substitutions in NLS of VP1 and VP2/VP3 Proteins Affect Virus Infection

**Figure 5:**
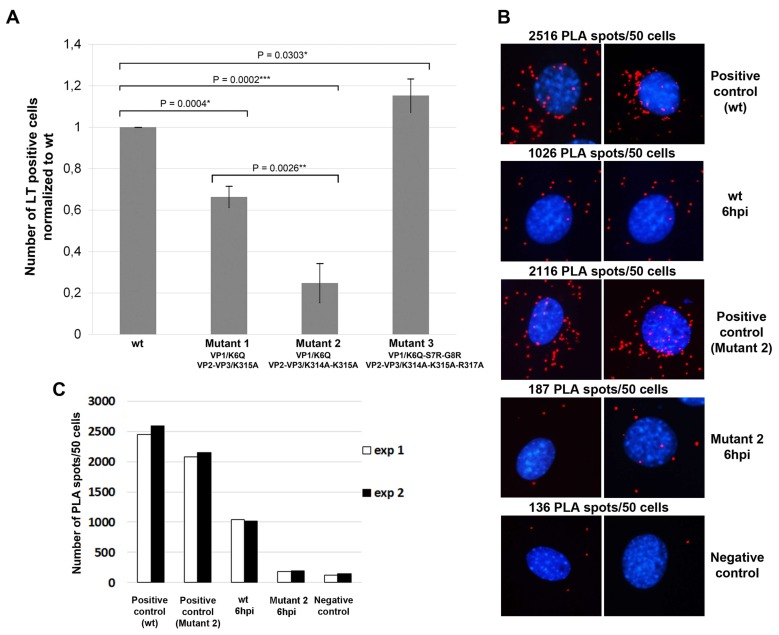
Effects of mutations in the NLS of VP1 and VP2/3 on viral infection and on binding of viruses to importin β1. (**A**) 3T6 cells were infected with wt or mutant viruses 1, 2, or 3 (using virus equivalent representing 1.5 × 10^4^ genomes per cell). Cells were fixed at 24 hpi and LT was stained by specific antibody. The presented data correspond to the mean of three independent experiments +/− standard deviation (s.d.). At least 300 cells were counted per experiment. Samples were compared by the *t*-test. *p* values are given and asterisks represent statistically significant differences (* *p* ≤ 0.1, ** *p* ≤0.01 *** *p* ≤ 0.001); (**B**) PLA assay was performed in 3T6 cells at 6 hpi with wt MPyV or the mutant 2 virus (200 virus particles per cell). For this experiment, we used primary mouse and rabbit antibodies against VP1 or mouse antibody against VP1 and rabbit antibody against importin β1. Next, the oligoprobes tagged anti-mouse and anti-rabbit antibodies were used. Red spots represent the products of amplification after oligonucleotide ligation. DNA was stained by DAPI. As controls, the infected cells were stained with two antibodies (mouse and rabbit), both against VP1 and non-infected cells were stained with anti-VP1 and anti-importin β1 antibodies. At the top of each image, the average numbers of the PLA spots quantified in two independent experiments are presented (for each experiment spots in 50 cells were counted). The pictures were taken 20× magnification; (**C**) The graph represents the mean values of two independent PLA experiments.

Furthermore, we evaluated the impact of the mutations of NLS of structural proteins on the delivery of virus genomes into the cell nucleus. We followed the percentage of infected cells producing early LT antigen. For this experiment, to better quantify the amount of virus used for infection, we measured the number of viral genomes using a qPCR approach. Virus amount, equivalent of 1.5 × 10^4^ genomes per cell, was used for the infection of the cells growing on coverslips. Cells were infected with wt or mutated viruses 1, 2, and 3, fixed at 24 hpi and stained with antibody against the LT antigen (Figure 5).

The results presented in Figure 5A revealed that the disruption of the NLS of VP1 and VP2/3 capsid proteins affected the ability of the virus to infect cells. Mutant 1 carrying one aa substitution in NLS of VP1 and one aa substitution in NLS of VP2/3 displayed 66% of infectivity in comparison with the wt virus. Infectivity of mutant 2, which had the same mutation in NLS of VP1 and two aa substitutions in NLS of VP2/3, reached only the 25% of the wt infectivity. Surprisingly, mutant 3, where three aa substitutions were made in NLS of VP2/3, while the NLS of VP1 was recovered, exhibited infectivity comparable with wt virus. The results show that disruption of NLS of both the VP1 and VP2/VP3 capsid proteins significantly decreases viral infectivity. To further prove this, we performed PLA assay to analyze the binding importin β1 to mutant virus 2. For this experiment, cells growing on coverslips were infected and fixed at 6 hpi. For comparison, cells infected with wt virus were used. PLA was performed as indicated in the Materials and Methods. The numbers of PLA spots were quantified in two independent experiments in 50 cells per experiment. Figure 5B shows representative pictures of the PLA spots and the graph (5C) a quantitative analysis. The average number of spots indicating a close proximity of virions and importin β1 per 50 cells decreased for the mutant 2 to 18% of the wt virus value. Actually, the value for the mutant 2 (187 spots) was very close to the number of spots obtained in the negative control (136) which represents the background observed in non-infected cells. Thus, the mutant 2 does not efficiently bind importin β1.

These findings suggest that (i) MPyV DNA delivery from the cytoplasm into the nucleus can be performed by the importins utilizing NLS of both VP1 and VP2/VP3 capsid proteins; and (ii) capsid proteins accompany the viral DNA to the nucleus during productive infection.

**Figure 6:**
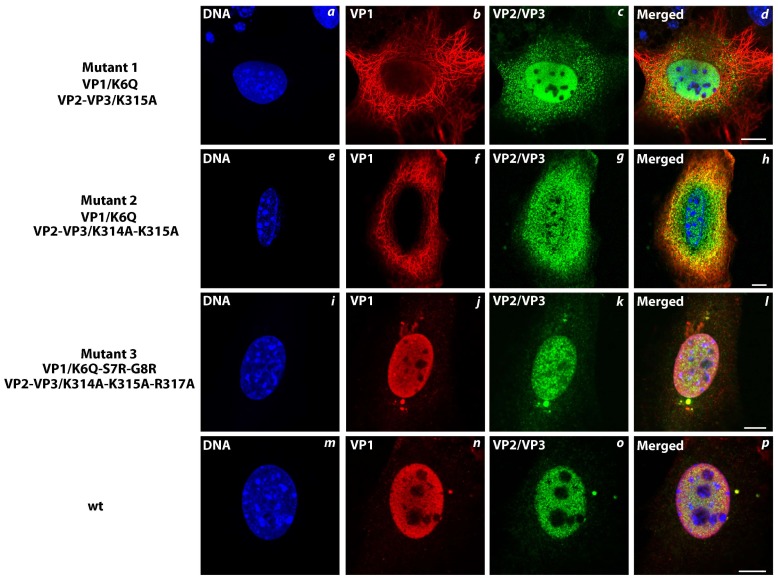
Effect of mutations in NLS of MPyV structural proteins VP1, VP2, and VP3 on their cellular localization during infection. Confocal sections of 3T6 cells infected with wt (**m**–**p**) or mutated viruses 1 (**a**–**d**), 2 (**e**–**h**), or 3 (**i**–**l**). Cells were fixed at 24 hpi and stained by antibodies against VP1 (red), VP2/3 (green), and DNA by DAPI (blue). Bars 5 µm.

We noticed changes in distribution of the newly synthesized capsid proteins in 3T6 cells infected with the mutated viruses (Figure 6). Mutant viruses 1 and 2, carrying the same version of mutated VP1, displayed localization of the VP1 predominantly in the cytoplasm (Figure 6b,f, respectively). In contrast, the wt VP1 protein was localized in the nucleus (Figure 6n). Thus, single point mutation introduced into the NLS of VP1 affected its nuclear transport. The VP1 of mutant 3 was localized, similar to the wt VP1, predominantly in the nucleus (Figure 6j), confirming the re-establishment of VP1 NLS function.

Results of the subcellular distribution of mutated VP2 and VP3 in cells infected with the mutant viruses were not as explicit as those for mutated VP1. VP2 and VP3 capsid proteins of mutant viruses 1 and 2 were localized in both the nucleus and cytosol (Figure 6c,g), while wt VP2 and VP3 proteins were localized in the nucleus (Figure 6o). Mutated VP2/VP3 of the mutant virus 3, where three basic aa of NLS were substituted by alanine, were transported into the nucleus in the most observed cells (Figure 6k). These results, together with high infectivity of mutant 3 suggest that functional NLS of VP1 in VP1-VP2/3 complexes is sufficient for delivery of the complexes into the cell nucleus. However, this observation is in contradiction with the observation made for the SV40 or BK polyomavirus [20,33], for which it was reported that functional NLS of the minor capsid proteins is important for the nuclear transport of the structural proteins. Therefore, to better understand the effect of the mutations on the nucleus targeting, we decided to analyze the distribution of the mutant and wt capsid proteins separately and in combination out of the context of infection.

### 3.5. Mutations Introduced into the NLS of the MPyV Capsid Proteins Affect Their Transport into the Nucleus during Their Individual Expression

**Figure 7:**
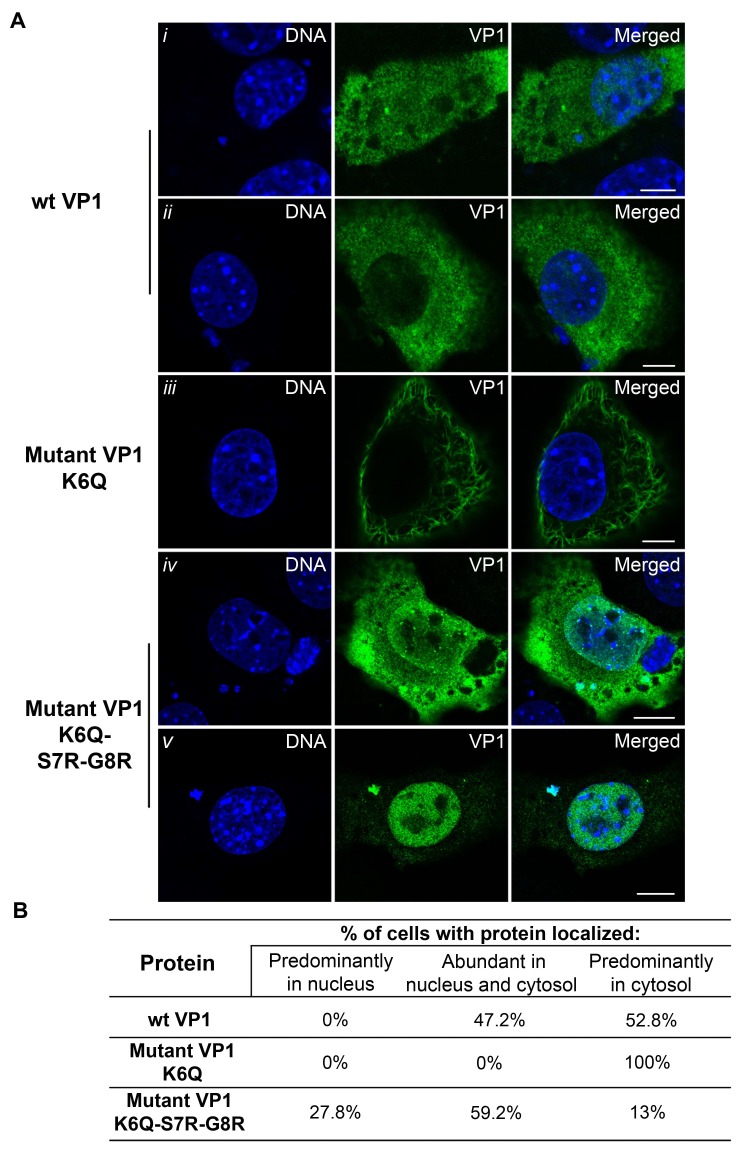
Subcellular distribution of wild type or mutated VP1 capsid proteins. Confocal sections of 3T3 cells expressing wt or mutated VP1 proteins. Cells were fixed at 24 hpt and stained with anti-VP1 antibody (green) and DNA by DAPI (blue) (**A**); For analysis of subcellular distribution of wt (**i**–**ii**) or mutated proteins (**iii**–**v**), 50 cells were examined for each variant. Bars 5 µm. Scores are given as percentage (**B**).

To produce transiently mutated capsid proteins, the same mutations described in Figure 3 were introduced into the plasmids expressing MPyV VP1 or VP2 genes. Localization of individually expressed wt or mutated variants of VP1 or VP2 in transfected 3T3 cells was followed by confocal microscopy. The cells expressing wt VP1 or VP1 mutant variants were analyzed at 24 h post transfection (hpt), while cells expressing wt VP2 or its mutated variants were analyzed at 5 hpt. The shorter time post transfection for the VP2 was used because the minor proteins could induce strong cytoxicity when expressed without the VP1 [43]. Confocal section representing the predominant phenotype of the cells expressing wt VP1 or mutant variants are shown in Figure 7A. For analysis, 50 cells for each individually expressed protein variant were analyzed for subcellular distribution. Three categories were considered: proteins located in nucleus, protein located in both nucleus and cytosol and proteins predominantly located in the cytosol. The score is presented as a percentage in the Figure 7B. We found that wt VP1 was localized in both the cytosol and nucleus (Figure 7i) in approximately half of the cell population (47.2%). In the second half of the transfected cells (52.8%), wt VP1 was preferentially localized in the cytosol (Figure 7ii). The mutated version of VP1 (K6Q) (which was present in mutant viruses 1 and 2) was exclusively cytosolic (Figure 7iii). The confocal data showed that the NLS of wt VP1 was not efficient enough for targeting the protein exclusively into the nucleus. Moreover, the substitution of the one basic aa (K6Q) in NLS completely abolished the import of VP1 into the cell nucleus. The VP1 mutant protein (which was present in virus mutant 3), where two additional substitutions were introduced (S7R and G8R) to restore the basic character of the NLS, similar to wt VP1, was in 59.2% of cells in both the nucleus and cytosol (Figure 7iv). However, the subpopulation of the cells that had a mostly cytosolic localization decreased to 13% and a new subpopulation of cells with VP1 preferentially located in the nucleus (27.8%) appeared (Figure 7v). Thus, the VP1 of mutant 3 seems to possess a stronger NLS than that of the wt virus.

**Figure 8:**
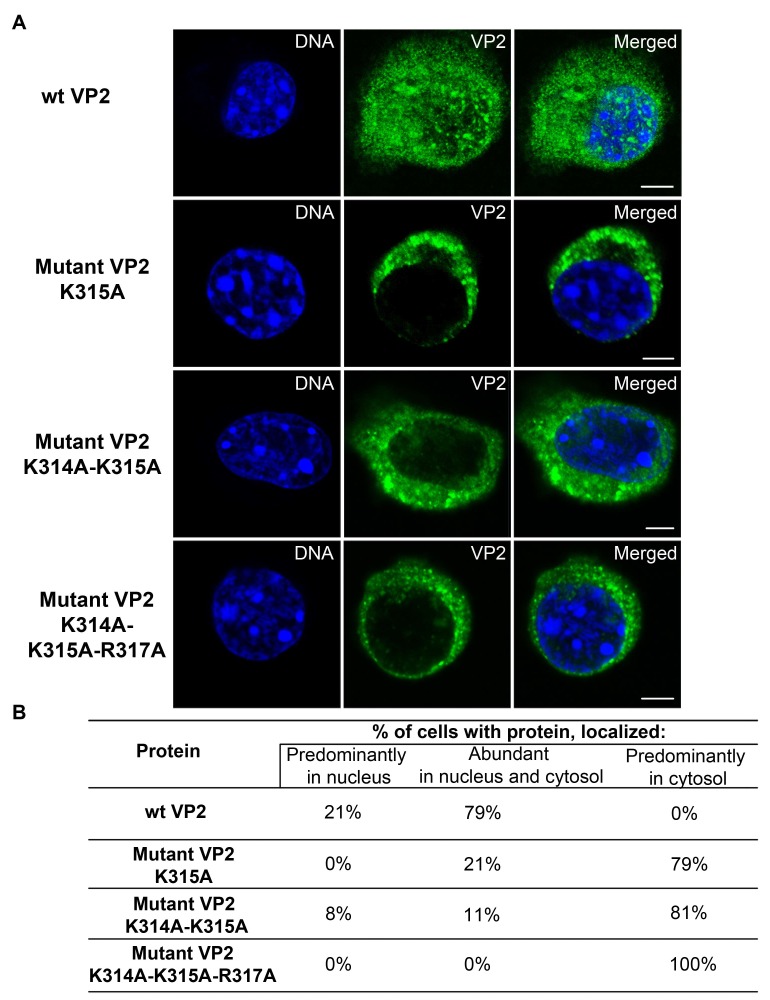
Subcellular localization of wild type or mutated VP2 proteins. Confocal sections of 3T3 cells expressing wt or mutated VP2 proteins. Cells were fixed at 5 hpt and stained by antibodies against VP2 (green) and DNA by DAPI (blue). Bars 5 µm (**A**); Scores are given as percentage (**B**).

Confocal section of cells representing the predominant phenotype of the cells expressing wt VP2 or its mutant variants are shown in Figure 8A. As above, 50 cells per wt or each mutated protein were analyzed for subcellular distribution of the protein. The relative scores are presented as percentage in Figure 8B. The wt VP2 protein was distributed in most of the transfected cells (79%) in similar proportion in the cytosol and nucleus, while in some cell subpopulation (21%), the protein was predominantly in the nucleus. Thus, similar to the NLS of VP1, the NLS of VP2 was not “strong” enough to ensure its efficient delivery into the nucleus in the majority of the cells when the protein was produced individually. VP2 protein mutated in one position (K315A) in the NLS, appeared predominantly in the cytoplasm in 79% of the transfected cells, while the protein was distributed in both the cytoplasm and nucleus in only 21% of cells. 

The VP2 protein mutated in two positions (K314A-K315A), localized prevalently in cytosol in 81% of the cells, and mutant protein VP2 with substitutions of three basic aa (K314A-K315A-K317A) was found in the cytosol in 100% of the cells.

Our data showed that substitution of one basic aa by a neutral one in the NLS region of VP2 significantly affected the nuclear localization of the VP2 protein, while the introduction of three mutations in VP2 NLS completely abolished the import of the protein into the cell nucleus.

### 3.6. Transport of the Major and Minor Capsid Protein Complexes into the Cell Nucleus Is Efficient even when NLS of either VP1 or VP2 Proteins Is Abolished

**Figure 9:**
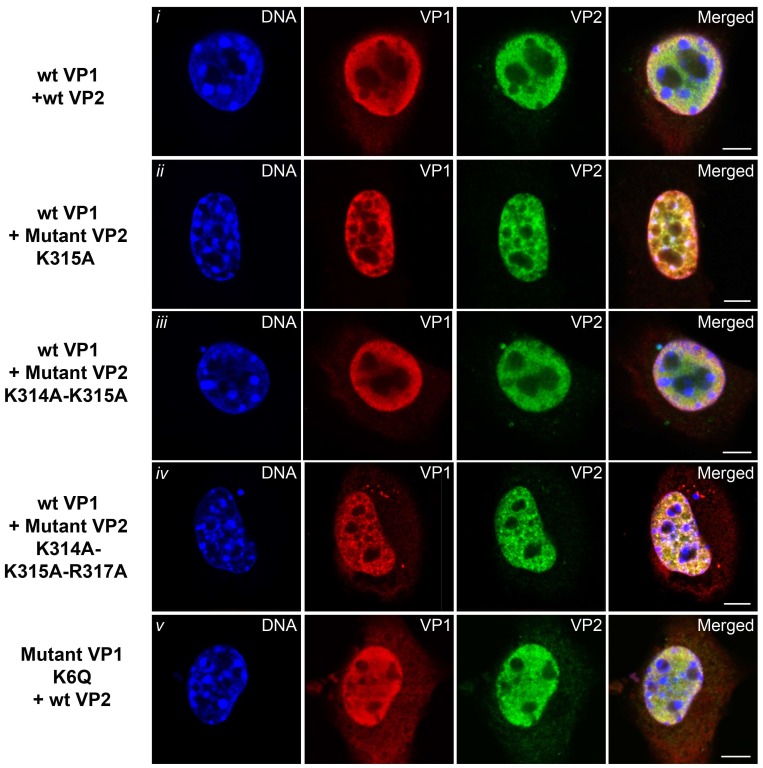
Nuclear entry of wt and mutant variants of the VP1 and VP2 proteins. Confocal sections of 3T3 cells co-expressing combinations of wt VP1 and wt VP2 (**i**) or wt VP1 and mutated variants of VP2 (**ii**–**iv**) or wt VP2 with the mutated variant of the VP1 (**v**). Cells were fixed at 24 hpt and stained with antibodies against VP1 (red) and VP2 (green). DNA was stained by DAPI (blue). Bars 5 µm.

We further investigated the nuclear translocation of complexes composed of wt VP1 and wt or mutant variants of VP2 and vice versa (mutated VP1 and wt VP2) after their co-production in 3T3 cells (Figure 9).

Images of representative confocal microscopy sections of cells expressing wt VP1 and wt VP2 are shown in Figure 9i, wt VP1 co-transfected with VP2 mutant variants with one, two or three aa substitutions in NLS are shown in Figure 9ii–iv, and co-transfected cells with VP1 mutated in NLS (K6Q) and wt VP2 is shown in Figure 9v.

The analysis of at least 50 transfected cells for each variant showed the efficient delivery of VP1 and VP2 into the nucleus for all co-expressed variants. The results revealed that complexes of VP1 and VP2 capsid proteins were efficiently transported into the nucleus even when just one of the proteins, VP1 or VP2, had a functional NLS. In contrast, the nuclear import of neither wt VP1 nor wt VP2 was efficient when these proteins were expressed individually (Figure 7 and Figure 8, first panels).

Taken together, our results indicate that MPyV virions mainly (or exclusively) use importin mediated transport for delivery of their genomes into the cell nucleus. The presence of both the major and minor proteins is required for efficient nuclear transport, apparently due to formation of a suitable conformation for importing binding.

## 4. Discussion

Mouse polyomavirus, a non-enveloped DNA virus, needs a sophisticated mechanism to overcome the cell membrane barriers to reach the nucleus. Years of study have revealed that the virus, being internalized into smooth vesicles, travels via early and late endocytic compartments into the ER from where it should be delivered into the cell nucleus [9,10]. Other studies have shed some light on the partial disassembly of virions in the ER and to the interactions of the virus with the ER membranes for translocation to cytosol [21,22,23,25,26,27,28].

In this study, we present evidence that once in the cytosol, MPyV uses α/β1 importin dependent transport as the main mechanism for the delivery of its genome into the nucleus for productive infection. We observed that virus association with importin β1 via NLS sequences of the capsid proteins VP1 and/or VP2/VP3 at early times post infection was time dependent. VP1 and MPyV genomic DNA were detected in the complexes with importin β1 at 3 hpi, their levels increased at 6 hpi, but decreased under the detection level at 8 hpi. The time of the VP1-importin interaction coincided with the time interval between the presence of the virus in the ER [44] and the time of the detection of the first MPyV early transcripts. According to Chen and Fluck, the first transcripts of the MPyV genome (detectable by RT PCR) appeared in the nucleus at 6 hpi [45].

Our data of immunoprecipitation and the PLA assay (Figure 1 and Figure 2, respectively) showed that only the minority of VP1 protein was in complex with importins, implying that after the virus appears in the cytosol, it is either quickly sorted to the nucleus or it is degraded by proteasomes. Our earlier data suggested that the successful delivery of the MPyV genomic DNA into the nucleus was a rare event [44]. In agreement, Nakanishi et al. [18] observed that despite an increase in the multiplicity of the SV40 virus used for infection, the amount of the VP1 and VP3 in immune-complexes precipitated with an antibody against importin α and β remained low. There could be several reasons for this phenomenon. First, not all virions that enter host cells are released into the cytosol. In our previous studies, we detected (at 5 hpi) only a subpopulation of the virus in the ER, while substantial portions of virions were found in late endosomal compartments, caveolin enriched vesicles, or in recycling endosomes [44,46]. Second, a fraction of the cytosolic, partially disassembled virions released from the ER, are likely to be degraded. Third, the transport of viral genomes or partially disassembled virions through the nuclear pore into the nucleus may encounter a series of barriers. For example, early studies into the trafficking of one of the non-enveloped DNA virus (human adenovirus) by using click chemistry and super resolution microscopy showed that a large pool of viral DNA was accumulated in the cytosol and did not enter the nucleus [47]. The failure to import the viral genomes into the nuclei can be caused by the improper docking of viral particles at the nuclear pores, incomplete translocation of viral cargo, by virus-induced stress, or by innate immunity mechanisms [48].

The analysis of infectivity of the MPyV mutated in the overlapping nuclear localization signals of VP1 and VP2 genes showed that VP1, or both VP1 and VP2/3 NLSs were involved in the delivery of MPyV genomes into the cell nucleus. Infectivity of mutant 1, where the NLSs of both VP1 and VP2/3 were weakened, was decreased by 34%, while the additional substitution in the VP2/3 NLS (K314A), which did not change any aa in VP1 (mutant 2), decreased infectivity of the virus (comparing with that of the wt virus) by 75%. This result underscores the importance of VP2/3 NLS. On the other hand, the additional mutations in mutant 3 (which introduced two basic aa to the NLS of VP1 thus recovering its strength, but completely abolished the NLS of VP2/3 by additional substitution) restored and even exceeded infectivity of the wt virus. Thus, this finding suggests that a strong NLS of VP1 is sufficient for the successful delivery of MPyV genomes into the cell nucleus. The infectivity results are in agreement with our data showing an inefficient transport of the newly synthesized capsid proteins in the cells infected with mutants 1 and 2, and an exclusive nuclear localization of the capsid proteins of mutant 3. All together, we showed that binding of structural proteins to importins is the main mechanism for viral delivery of genomes to nucleus. The question remains, how some mutant 2 virions with destroyed NLS of the structural proteins reach the cell nucleus to ensure residual infectivity (20% of that of the wt virus). One possibility is that the virus appeared in the nuclei during mitosis of subpopulation of cells. We cannot exclude also some alternative pathway(s), for example, direct entry of virions to the nucleus from ER. 

Even more puzzling question is where the mutated virus assembles. We performed EM of ultrathin sections of cells transfected with mutant 2 genomes at late times post-transfection (after 48 h) and found sporadic, usually single cluster of viral particle progeny in the nuclei of some transfected cells (Figure S2A–C). That means that mutant 2 capsid proteins were somehow at very late times delivered into the cell nucleus. The process must be very inefficient judging by the presence of a small cluster of virions beneath the nuclear membrane (while the wt virus fulfills the space throughout the nuclei by virus progeny at late times post-infection or post-transfection by genome DNA—see Figure S2D–G). The mechanism responsible for appearance of mutated capsid proteins in the nucleus is unclear and will be further explored. One possibility is that subpopulation of complexes formed by mutated capsid proteins accumulating in the cytosol can be partially degraded and the minor capsid proteins released from VP1 complexes can attack by their hydrophobic domains nuclear membrane, causing its local damage. Viroporin-like properties of the minor capsid proteins (when they are not bound to VP1 pentamer) were described previously [43]. 

The observation made for SV40 or BK polyomavirus [20,33] suggested that functional NLS of the minor capsid proteins VP2 and VP3 is important for the nuclear transport of structural protein complexes into the nucleus. Nakanishi et al. showed that VLPs (composed of VP1 with intact NLS and VP3 with disrupted NLS) were impaired in delivering SV40 DNA to the nucleus and, unlike its wild-type counterpart, the internalized mutated VLPs were not recognized by importins [33]. Similarly, the mutational analysis of the NLS of capsid proteins of human BKPyV suggested that BKPyV enters the cell nucleus by using the NLS of the minor capsid proteins as the nuclear import signal [20]. In contrast, our experiments studying infectivity of mutated MPyV viruses suggest that MPyV can use the NLS of VP1 only or, cooperatively, both VP1 and VP2/VP3 NLSs.

Our present study and our earlier reports showed that the nuclear import of individually expressed capsid proteins of the MPyV is poor [25] and Figure 8 of this manuscript. This is in contrast with the similar experiments performed with capsid proteins of BKPyV or SV40, where VP2 or VP3 were found exclusively in the nucleus [20,30] and our data when we analyzed by confocal microscopy the subcellular localization of the BK minor proteins [49]. We should point out that there are differences in the common *C*-terminal sequences of the minor proteins VP2 and VP3 between primate (BKPyV, JCPyV, SV40) and mouse polyomaviruses. In contrast to MPyV, an additional track of basic aa is present at the *C*-terminus of VP2/VP3 of primate polyomaviruses. This may contribute to the strength of the NLS of their minor proteins. Indeed, the NucPred program predicted scores for the strength of NLS of the minor capsid proteins (on the scale 0.1–1.0) are: 0.58 for BKPyV, 0.51 for JCPyV, 0.61 for SV40, but only 0.14 for the MPyV (Table S1). Thus, these remarkable differences in NLS strength may account for preferentially utilizing the NLS of the VP2/VP3 for the nuclear entry of primate polyomaviruses.

The predicted values of NLS strength of the VP1 are comparable for both the MPyV and primate polyomaviruses and range between the values 0.27–0.38. While wt MPyV VP1, produced individually (NLS strength 0.30), is found mostly in the cytosol, the VP1 of SV40 (with prediction of the NLS strength 0.38) is mostly targeted to the nucleus [30]. A different situation is observed with the Merkel cell carcinoma polyomavirus (MCPyV). Similar to the MPyV, its VP2 has weak NLS (0.18) which does not ensure the nuclear localization of VP2 protein when expressed without VP1. However, relatively strong NLS of the VP1 (0.45) targets this protein (and also the VP2, when it is in complex with VP1) to the nucleus [50]. Interestingly, the co-expression of mutated and wt MPyV capsid proteins revealed that mutated VP2 is able to help to transport both VP1 and mutated VP2 into the nucleus, even when its NLS is destroyed by three substitutions (NucPred score 0.04) and, vice versa, the complex of the mutated VP1 (NucPred score 0.17) and wt VP2 is also targeted into the nucleus, despite the fact that both proteins, when produced individually, had predominantly cytosolic location. This observation suggests that conformation of the capsid proteins in complex substantially enhances their nuclear import and importin α binding. One functional NLS, either that of VP1 or VP2/3 seems to be sufficient for the delivery of MPyV VP1-VP2 complexes into the nucleus, although none of these proteins is delivered into the nucleus separately. Similar observation was made for the mutated capsid proteins of SV40 [30].

Seven isoforms of importin α were identified in humans and six in mice. Their functional diversification occurs in all multicellular organisms [51]. In vivo, these isoforms are grouped into three subfamilies known as α1, α2, and α3 and these subfamilies display substantial substrate specificity which is not always proved in in vitro experiments [52]. Köhler et al. found that most substrates tested were imported by all isoforms of importin α with a similar efficiency [53]. However, substantially different substrate preferences of the various importin α isoforms were revealed when two substrates were offered simultaneously. In pilot experiments using the PLA assay, we found that MPyV VP1 interacted with the representatives of all importin α subfamilies. It cannot be ruled out that different isoforms can preferentially bind separate capsid proteins, capsid protein complexes, or partially disassembled virions. Interestingly, it has recently been shown that for the binding of importin α3, the three-dimensional structure context of NLS is determining rather than the aa sequence of NLS [54].

## Supplementary Materials

The following are available online at http://www.mdpi.com/1999-4915/10/4/165/s1, Figure S1: DNA sequence chromatograms of wt or mutant viruses. Figure S2: Nuclear assembly of the Mutant 2 virus and the wt virus. Table S1: Comparison of NLS signals strength in members of polyomavirus family.
