# Supplementary material for: Interaction of the Mouse Polyomavirus Capsid Proteins with Importins Is Required for Efficient Import of Viral DNA into the Cell Nucleus
Source: Viruses. 2018 Mar 31;10(4):165. doi: 10.3390/v10040165 (PMC5923459; doi:10.3390/v10040165)
Supplement: Supplementary file 1 [file viruses-10-00165-s001.pdf]

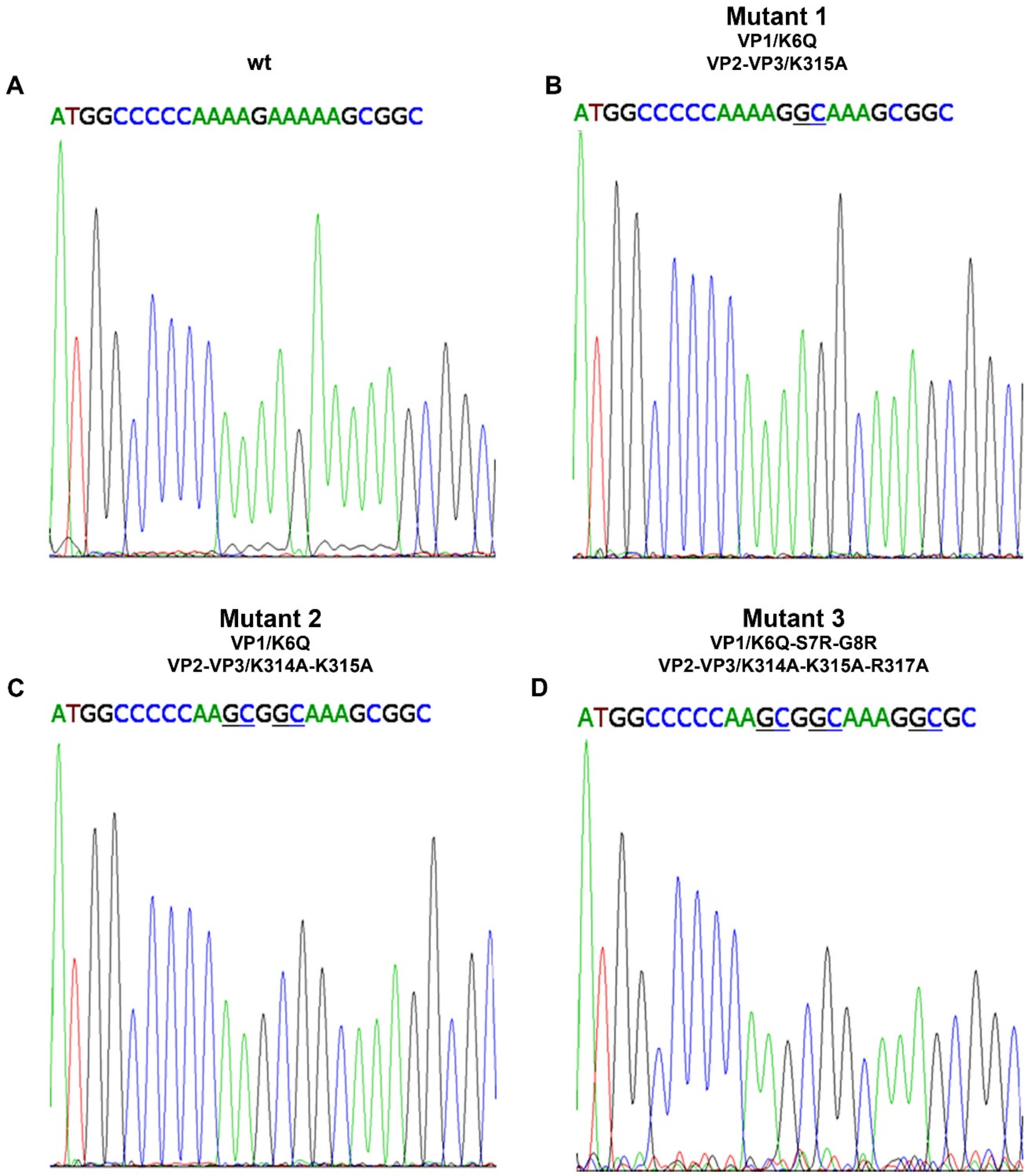

Figure S1. **DNA sequence chromatograms of wt or mutant viruses.** To verify the mutations introduced in to the genomes as well as to detect possible revertants we isolated DNA from virus preparations and performed sanger sequencing reaction by using The BigDye® Terminator v3.1 Cycle Sequencing Kit's robust (Thermofisher). The primers used were selected to amplify terminal part of MPyV VP2 gene (VP2 gene has 960 nucleotides). The nucleotides 929 to 952 are displayed in the figure. The introduced mutations are underlined.

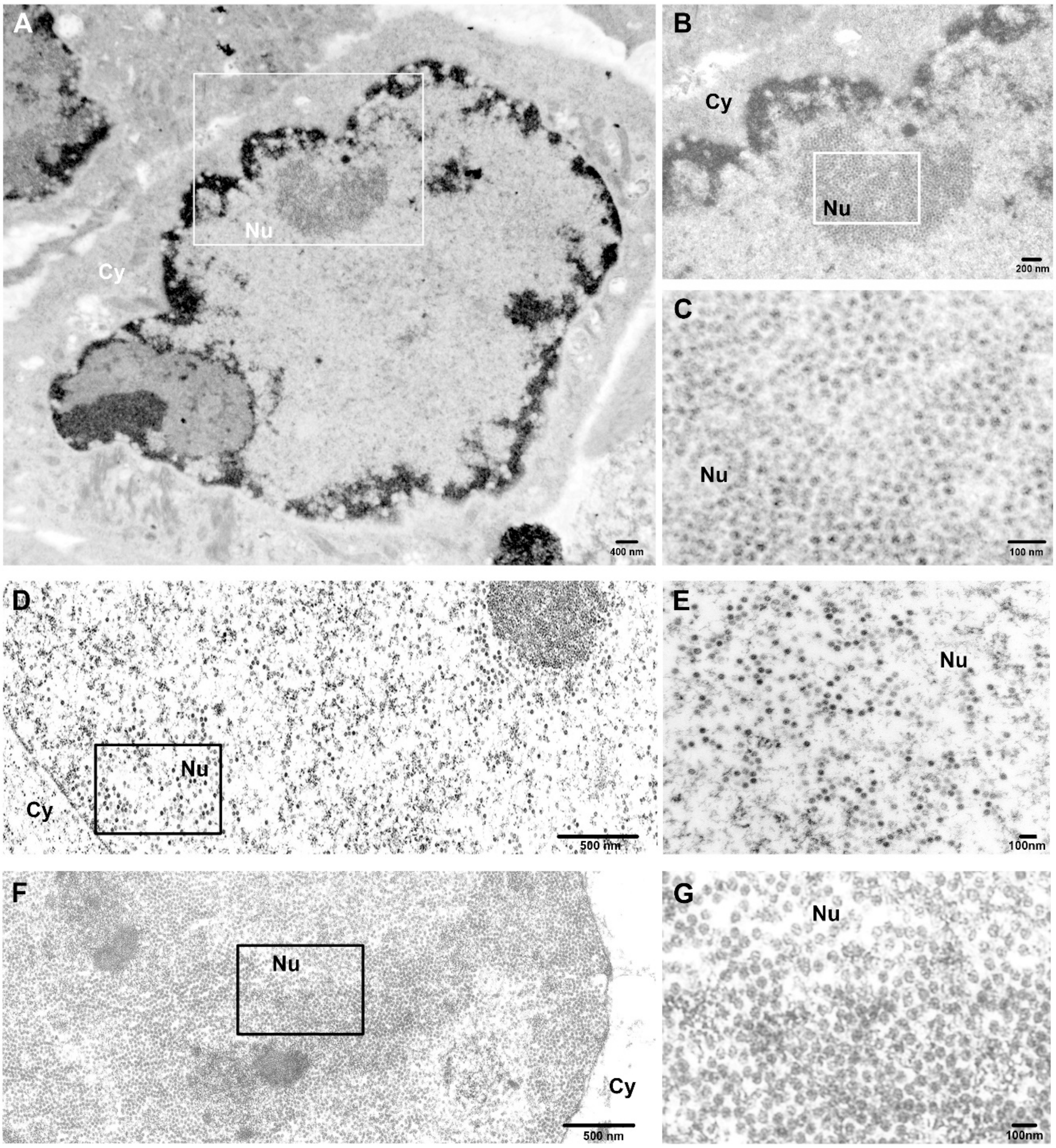

Figure S2. Nuclear assembly of the Mutant 2 virus and the wt virus. Electron microscopy of ultrathin sections of 3T6 transfected with Mutant 2 genomes cells (A-C) or wt genomes (D, E) or infected with wt virus (F, G). Cells were fixed and processed after 48 h. Cy, cytoplasm; Nu, nucleus.

Table S1. Comparison of NLS signals strength in members of Polyomavirus family. Sequences of VP2 and VP1 proteins of the different members of polyomavirus were analyzed by NucPred Program available online [35]. proteins with score between 0.10 and 1 are predicted to spend some time in the nucleus. The higher score most likely indicates that the protein is located at the nucleus.

Polyomavirus species (NCBI Reference Sequence)	**NLS score NucPred Program**	
	**VP2 Protein**	**VP1 protein**
SV40 (NC-001669.1)	0,61	0,38
JCPyV (J02226)	0,51	0,27
BKPyV (NC-001538.1)	0,58	0,37
**MPyV (J02289.1)**	**0,14**	**0,30**
MCPyV (NC-010277.2)	0,18	0,45
